# *Oestrus ovis* in Ecuador: Importance in the Andean sheep farming

**DOI:** 10.14202/vetworld.2019.522-526

**Published:** 2019-04-14

**Authors:** Gabriela Ortega-Muñoz, Nivia Luzuriaga-Neira, Richard Salazar-Silva, Richar Rodríguez-Hidalgo

**Affiliations:** 1 Department of Parasitology, Medicine, Veterinary and Zootechnic Faculty, Central University of Ecuador, EC170521, Quito, Ecuador; 2 Wildlife Conservation Research Unit, Central University of Ecuador, EC170521, Quito, Ecuador; 3 Parasitology Unit, Public Health and Zoonosis Research Institute, Central University of Ecuador EC170521, Quito, Ecuador

**Keywords:** Ecuador, Ecuadorian highlands, oestrosis, *Oestrus ovis*, sheep

## Abstract

**Aim:**

This study aimed to determine the prevalence of *Oestrus* *ovis* in sheep meant for meat commercialization in the main slaughterhouse of the country.

**Materials and Methods:**

Between October 2015 and December 2015, we assessed the occurrence of *Oestrus* myiasis in the main slaughterhouse localized in Quito. In total, 80 sheep heads were randomly inspected and necropsied. Larvae were removed from nasal cavities and paranasal sinuses and cleaned. ANOVA (generalized linear model) was used to estimate the relationship between sex, age, and place of origin and presence or absence of parasite larvae.

**Results:**

Morphological identification confirmed that 19% (15/80) of the examined animals were positive for *Oestrus ovis*; from the positive cases, 21% were young animals <12 months old. We found that statistical differences by animal sex, males, were most infested 93% (14/15) than females 7% (1/15). Larvae’s L2 were more abundant than other stages (62 of the total 149). 14 of the infested animals were from the Andean places at > 2500 meters above sea level (m.a.s.l.), and only one case from the coastal region at 250 m.a.s.l. with tropical environmental conditions.

**Conclusions:**

Our results showed evidence of the presence of myiasis caused by *O. ovis* in Andean and coastal places in Ecuador and its adaptation to different environmental conditions from that reported previously in temperate regions from Europe and Africa.

## Introduction

The sheep bot fly *Oestrus ovis* (*Diptera*: *Oestridae*) is a cosmopolitan and obligate parasite in domestic and wild ruminants, mainly founding sheep and goats and eventually in some wild species [1,2] and humans [3-6]. Oestrosis causes low mortality. However, it produces high mobility in infected animals. Its signs and symptoms cause a negative effect on the herd, affecting long-term productivity with severe consequences on livestock production [7].

Oestrosis is present in different regions and habitats and has been reported worldwide, i.e., in the Mediterranean countries of Europe and Africa [8,9], Asia, and India [1,5]. In America, this parasite was found from California to the Patagonian regions, including Central America [10-12], as *O. ovis* activities appear to be accustomed to high temperatures (25-28°C), strong solar radiation (116-838 Wm^−2^), and a wide range of the relative humidity (65-85%) [13]. Besides, a high prevalence was found in tropical sub-humid sites where temperature, annual rainfall, and humidity were 26°C, 900 mm, and 60-80%, respectively [14]. Furthermore, in tropical regions, most oestrosis risks were documented in the sites with high temperatures and lower rainfalls [15]. So far, oestrosis has not been reported in Ecuador or the Ecuadorian highlands.

In spite of sheep bot fly being well-known by Andean Ecuadorian farmers, there is no information about their prevalence and incidence, or the economic losses caused by its presence. On the other hand, ovine livestock is becoming important in both highland and tropical regions; hence, the risk of infested animals by *O. ovis* must be evaluated.

This study aimed to determine the prevalence of *Oestrus* *ovis* in sheep meant for meat commercialization in the main slaughterhouse of the country.

## Materials and Methods

### Ethical approval

No ethical approval was needed for this study. None of the animals were used experimentally, and collection of the specimens was carried out after slaughter.

### Sample collection

In total, 80 sheep heads were inspected at the Metropolitan Slaughterhouse in Quito (located at: Latitude −0.318794 and Longitude −78.563794) between October 2015 and December 2015. Around 300 sheep are slaughtered per month; hence, approximately 13% of ovine were selected randomly. Due to slaughterhouse’s logistic, five sheep’s heads were sampled each Tuesday and Thursday. Data from the sanitary certificate from each slaughtered animal were obtained to analyze geographical origin, sex, and age (Table-1). In addition, climate data were collected from the annual meteorological reports published by the National Institute of Meteorology and Hydrology [16].

**Table-1:** Larval stages of *O. ovis* recovered from the positive animals slaughtered in the Ecuadorian highlands.

Positive animal			Larvae stage				Animal location		Meteorological data			
			
Head	Age	Sex	L1	L2	L3	Total	Origin	Coordinates (Latitude and longitude)	Humidity (%)	Precipitation (mm^3^)	Altitude (masl)	Temperature (°C)
1	Adult	M	0	15	7	22	Ambato	−1.24908, −78.61675	76	403.4	2515	15
2	Adult	M	0	9	6	15						
3	Young	M	0	0	3	3						
4	Young	M	0	0	4	4						
5	Young	M	0	0	5	5						
6	Young	M	4	13	2	19						
7	Young	M	0	0	11	11						
8	Adult	M	2	1	4	7						
9	Adult	M	0	1	1	2	Guayaquil	−2.16667, −79.9	80	966.6	250	26
10	Adult	F	5	6	0	11	Latacunga	−0.93333, −78.61667	75	347.3	2785	13
11	Young	M	10	5	0	15	Rumiñahui	0.33405, −78.45217	79	555.1	2500	15
12	Adult	M	18	9	0	27	Salcedo	−1.03333, −78.56667	75	1000	2683	14
13	Adult	M	2	1	0	3						
14	Young	M	0	2	1	3						
15	Adult	M	0	1	1	2	Saquisili	−0.83333,−78.66667	75	1000	3340	13
Total		15	41	63	45	149						

Age: Young=0-12 months, Adult=>12 months, Sex: M=Male, F=Female, Larval stages: L1=Stage 1, L2=Stage 2, L3=Stage 3, *O. ovis*=*Oestrus ovis*

### Sample processing

**Figure-1:**
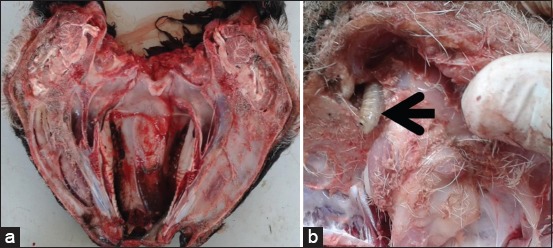
Examination method of the presence of *Oestrus ovis* in individuals destined for slaughter, in a public slaughterhouse of the Metropolitan district of Quito. a= cross section of the head; b= extraction of *Oestrus ovis*.

At the slaughterhouse, heads were inspected as described by Bowman [17] (Figure-1). Briefly, the heads were dissected by cutting along their longitudinal axis to expose nasal cavities and paranasal sinuses, which were subsequently cleaned with distilled water. Larval samples were collected as described by Yilma and Dorchies [18].

### Larvae analysis

Collected larvae were coded and placed in a container with 70% of ethanol caps and transported to the parasitology laboratory of the Veterinary Faculty of the Central University of Ecuador. Morphological identification was performed by direct observation using a stereoscope and dichotomous keys; the larvae were identified as described by Moya *et al.*, [19].

### Statistical analysis

Associations between the presence of the parasite and animal origin, sex, and age were estimated using ANOVA (generalized linear model) with binomial distribution in R statistic free software version 3.2, package MASS [20]. We consider dependent variable the presence or absence (0=absence and 1=presence), and the independent variables: Animal origin (n=7), sex (M=male and H=female), age (<1, > 1-year-old), and age of the sheep were recorded from the certificate sanitary.

## Results

**Figure-2:**
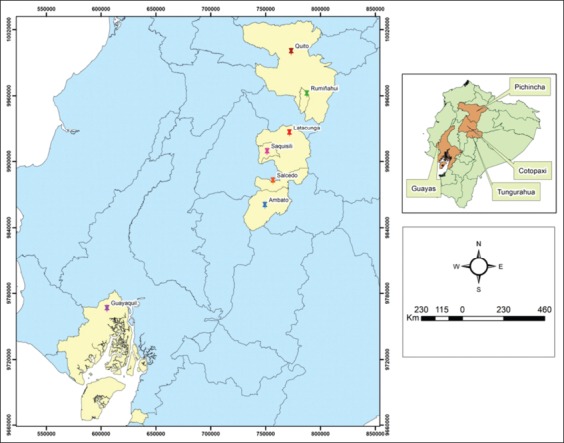
The geographical location of the places of the origin sheep infected with *Oestrus ovis* parasite, in the Coastal and Andean regions of the Ecuador.

From 80, heads inspected 15 (19%) were infested with *O. ovis* larvae. Moreover, among positive cases, males were most infected than females 14 (93%) and 1 case (7%), respectively. 14 sheep came from Andean region farms located at >2500 meters above sea level (m.a.s.l.). with low temperatures and dry areas and one infested animal came from the coastal area at 50 m.a.s.l. with high temperatures and humidity annual averages (Table-1). Geographical location of positive cases is graphed in Figure-2, i.e., 53.3% from Ambato, 20% from Salcedo, 20% from Saquisilí, Latacunga, and Rumiñahui at the highlands, and 6.6% from Guayaquil in the Coastal region (Figure-2). Most positive cases were from Andean sites with minimal differences in temperature (14-15°C) and humidity (75-76%) annual averages.

No statistical differences were found for age and origin; however, animals aged 3 months-1 year had a higher percentage of infestation (21%) than animals older than 1 year (17%). Statistical differences (<0.05) were found for sex, i.e., males, with 23% (14/15), while females reached 5% (1/15) (ANOVA: F_40.8_=1, p=0.043) (Table-2). On the other hand, in total, 149 larvae (42% [L2]; 30% [L3]; and 28% [L]) were collected from 15 infected heads (Table-1). The mean larval burden per sheep was 5.4 larvae. The average larvae stages did not differ (p>0.05) among groups (L1=2.73±5.07; L2=4.20±5.07, and L3=3.00±3.20).

**Table-2:** Differences of sex, age, and site on the presence-absence of *O. ovis* in sheep (n=80) from six sites of Ecuador. The results are from GLM with binomial distribution (95% of probability).

Coefficients	DF	DR	RD	F	p<0.05
Sex	1	40.791	73.133	40.791	0.04342*
Age	1	0.0191	68.994	0.0191	0.89010
Origin place	7	33.811	65.613	0.4830	0.84765

Origin places: Ambato, Guayaquil, Mejía, Latacunga, Rumiñahui, Salcedo, and Saquisilí. Males sheep were most infected than females (p<0.05)

* show statistical differences insignificant level of 0.05. DF=Degree of freedom, DR=Deviance residual, RD=Residual degree, F=F statistic, *P*=p-value. Age: Young=0-12 months, Adult=>12 months, sex: M=Male. GLM=Generalized linear model, *O*. *ovis*=*Oestrus ovis*

## Discussion

Our findings showed 19% of the prevalence of *O. ovis* in the highlands of Ecuador. Previously, other studies carried out in South America showed a prevalence of oestrosis of 13.7% and 16.9% in Brazil [10,21], 60% in Chile [12], and 33.4% in Mexico [14]. All these studies were carried out in different geographical and ecologic zones with different climatic conditions to our study. According to Abo-Shehada *et al*. [22], Cepeda-Palacios and Scholl [13], Papadopoulos *et al*. [23], and Paredes-Esquivel *et al*. [24], the *O. ovis* flneeds specific conditions for development, i.e., when temperatures are between 25 and 28°C and the solar radiation is between 116 and 838 Wm^−2^ and these conditions are mainly present in temperate regions in the spring and summer season. In addition, the authors documented higher oestrosis risk in sites with higher temperature and lower rainfall [25]. Ecuador is a tropical country where different ecological niches are present due to the Andean Cordillera. Most infested animals stem from the highlands, where temperatures and solar radiations are unlike those from temperate regions [16].

In our study, no statistic difference (p>0.05) was found between the age and sex of animals that were oestrosis positive, probably due to the random selection at the slaughterhouse or a similar trend in the management in sheep livestock. Other studies reported different results and mentioned that adults or even females are more affected than young or male sheep [10,26,27]. Unexpectedly, one positive animal came from the coastal region where myiasis is supposed to be absent; however, further studies are needed to demonstrate the prevalence and importance of oestrosis in the tropical areas of Ecuador.

The number of larvae and larval stages found in this study were near to Caracappa *et al*. [27], i.e., on average 5.4 larvae per infested animal, in Sicily-Italy. In another study carried out on the Balearic Islands, a higher percentage of L1 was reported [24]. Although all these which indicate an active flying activity of adult *O. ovis* flies on the sites with Mediterranean climate, our results demonstrated fly activity at the high altitudes of Andes (>2500 m.a.s.l.), where only two seasons are evident, i.e., rainy (October-April) and dry season (May-September) according to the reported case published by Hoyer *et al*. [28]. Moreover, sheep livestock is important in the Andean Highlands from Colombia to Argentina, where the population was estimated to be around 57 million sheep [29]; hence, it is most likely that this parasite is affecting farms and causing huge loses not yet estimated in livestock.

According to our observation, this study is the first record in sheep which shows the evidence and importance of oestrosis in environments >2500 m.a.s.l. at Andean region in South America. Most of the results found on the epidemiology of *O. ovis* were reported from temperate regions such as Spain, Italy, Turkey, and France [8,18,26,27], where infested cases are seen at low altitudes (e.g., 250-1725 m.a.s.l.) and temperatures >30°C in the summer season. This, in contrast with our study region, shows that *O. ovis* fly is adapted to high altitudes and dry habitats.

## Conclusion

Our study demonstrates the presence of *O. ovis* at a high altitude in six different locations in Ecuador; five of them from the Andean Region, and one from the Coastal Region where tropical environmental conditions predominant. Furthermore, studies are recommended to assess the prevalence, incidence rate, risk factors, and spatial-temporal distribution in Andean and Tropical regions in Ecuador. Although the presence of the disease has been known by farmers, the understanding of *O. ovis* epidemiology should be improved.

## Authors’ Contributions

NL and RR conceived the study and participated in its design and coordination. RS and GO carried out the sampling and the analysis. RR and NL wrote the manuscript, and all authors read and approved the final manuscript.
